# Supplementary material for: Acoustic sorting of microfluidic droplets at kHz rates using optical absorbance
Source: Lab Chip. 2022 Dec 1;23(1):195–202. doi: 10.1039/d2lc00871h (PMC9764809; doi:10.1039/d2lc00871h)
Supplement: LC-023-D2LC00871H-s001 [file LC-023-D2LC00871H-s001.pdf]

Electronic Supplementary Material (ESI) for Lab on a Chip.
This journal is © The Royal Society of Chemistry 2022

Supplementary Information
Acoustic sorting of microfluidic droplets at kHz rates using optical absorbance

Footprint of the System

Images of the experimental setup showing the outer footprint of the system, Fig.1, and the footprint of the microchannels as a microscope image of the device, Fig. 2.

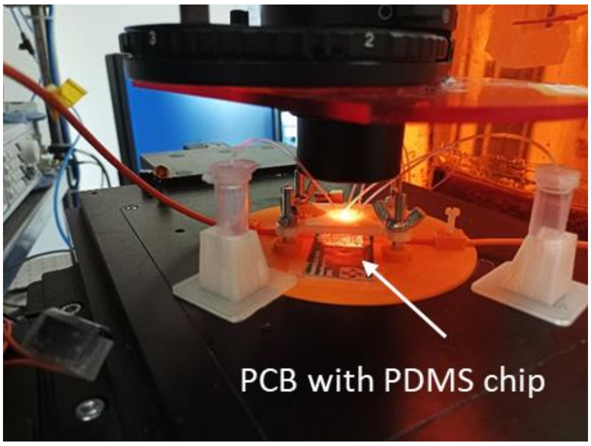

Fig. 1 Outer footprint of the sorting setup showing the microfluidic chip in a chip holder on the microscope stage

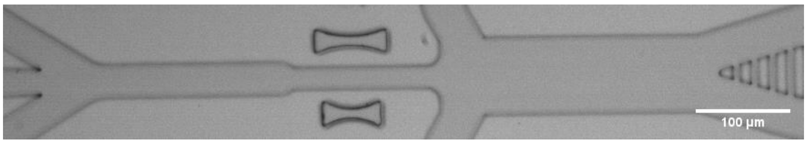

Fig. 2 Inner footprint of the microflu*i*dic sorting chip showing the channel structure from the spacing point to the sorting poitn at the bifurcation of the two outlet channels. This image corresponds with the device schematic describing the functional regions of the device in Fig. 1 of the paper. The distance from the spacing to the tip of the bifurcation is ca. 700 µm.
